# Optimizing the Utilization Rate and Performance of 3D-Printed Mortar with Dual-Size Recycled Sand

**DOI:** 10.3390/ma19071478

**Published:** 2026-04-07

**Authors:** Jie Huang, Xinjie Wang, Quanbin Shi, Pu Yuan, Minqi Hua

**Affiliations:** 1 School of Urban and Rural Construction, Taizhou Polytechnic College, Taizhou 225300, China; huangjie@tzpc.edu.cn (J.H.); sqb.tz@163.com (Q.S.); 2 School of Urban Construction, Changzhou University, Changzhou 213164, China; yuanpucczuedu@163.com; 3 School of Civil Engineering & Architecture, Wuhan University of Technology, Wuhan 430070, China; hmq@whut.edu.cn

**Keywords:** 3D printing, material balance method, tilt angle, interlayer splitting and bonding strength, Vickers hardness

## Abstract

To enhance the utilization rate and mechanical performance of recycled sand (RS) in extrusion-based 3D printing, this study investigates the influence of varying incorporation ratios of RS across two particle size fractions: 0.075–1.18 mm (RS01) and 1.18–2.36 mm (RS12). The RS utilization rate was determined via the material balance method, while microstructural mechanisms were analyzed using scanning electron microscopy and Vickers microhardness testing. The results indicate that: a combination of 75% RS01 and 25% RS12 achieves the maximum RS utilization rate of 84.3%. At an RS12/RS01 ratio of 1:3, the printed specimens exhibit the smallest tilt angles in bidirectional buildability tests, measuring 7.6° and 7.2°, with corresponding tan θ values of 0.066 and 0.063. Compared to mortar with 100% RS01, this optimized mixture yields average increases of 36.5% in compressive strength, 40.7% in flexural strength, and 6.8% in interlayer splitting strength. Analysis of variance indicates that different particle size combinations have a significant effect on the mechanical properties. Microhardness analysis reveals that the combination of 75% RS01 and 25% RS12 achieves a minimum interfacial transition zone width of 46 µm. Utilizing larger-particle-size RS in 3D printing effectively enhances its utilization rate while maintaining satisfactory printability and mechanical properties.

## 1. Introduction

Accelerated urbanization and the renovation of existing buildings have led to the generation of substantial construction and demolition waste, which has become a global environmental issue [1,2]. How to efficiently recycle construction waste represents one of the major challenges in achieving sustainable development in the construction industry [3,4]. Three-dimensional (3D) printing technology, as an emerging intelligent construction method, has attracted widespread attention due to its potential for digitization, automation, and reduction of material waste. Particularly in the field of concrete materials [5], extrusion-based 3D printing offers a new pathway for high-value utilization of construction and demolition waste [6,7]; however, the low utilization and high consumption of this waste are still concerning [8].

The application of recycled aggregates in 3D-printed concrete not only facilitates the effective utilization of construction and demolition waste [9], but also reduces the reliance on natural aggregate resources [10]. Its use in 3D printing mortar not only enables the reuse of construction waste but also supports the objectives of sustainable construction [11,12]. Although the application of recycled sand (RS) in conventional concrete has been extensively studied, its efficient utilization in extrusion-based 3D printing remains a significant challenge [13,14]. The 3D printing process imposes strict requirements on the printability of materials, including extrudability and buildability. However, RS inherently exhibits high porosity, water absorption [15], and adhered old cement paste, which significantly alter the rheological properties and hydration process of fresh mortar [16,17]. In practical applications, these characteristics tend to cause poor fluidity, reduced extrusion performance, and insufficient mechanical strength after hardening [18]. Therefore, the replacement ratio of RS is generally low [19], and fibers or cementitious materials are required to enhance performance. Zou et al. [20] found that incorporating an appropriate amount of sodium gluconate into 100% recycled mortar could not only satisfy the printability requirements for 3D printing but also resulted in an excellent printing window and higher green strength. Subsequently, Xiao et al. [21] introduced polyethylene fibers to enhance both the printability and mechanical properties of 100% RS-based 3D-printed materials, shifting the failure mode of the specimens from brittle to ductile. Raghed et al. [22] fully replaced NS with RS and observed no significant differences in mechanical strength between printable mortars based on either natural or recycled sand.

Studies have indicated that the particle size distribution of aggregates is one of the factors influencing the flowability and mechanical strength of mortar [23]. By optimizing the combination of aggregates with different particle sizes, a tighter packing density can be achieved, reducing water demand and thereby improving the workability of the mixture and enhancing its microstructure [24]. In conventional concrete, several studies have utilized sands of varying sizes to enhance overall fluidity and mechanical performance [25]. Li et al. [26] investigated the effect of fine aggregate gradation on the rheological properties of mortar and found that the influence of gradation became more pronounced with the increase in the volume fraction of fine aggregates. Ghazal et al. [27] employed X-ray computed tomography to characterize the aggregate gradation in hardened concrete, demonstrating that different gradations significantly affect their workability. Chen et al. [28] investigated the effect of coarse aggregate gradation on the workability of 3D-printed concrete and found that continuously graded concrete exhibited better performance in terms of fluidity retention.

Although previous studies have demonstrated the feasibility of fully replacing natural sand with recycled sand in 3D-printed mortar, the particle size of the recycled sand employed in most cases is typically limited to the range of 0.075 mm to 1.18 mm. The utilization of recycled sand with other particle sizes in 3D printing remains to be further explored. Moreover, relying solely on fine recycled sand may limit the efficient use of available resources. To date, research on the combined effects of recycled sands with varying particle sizes on the printability and mechanical properties of 3D-printed mortar remains limited. In particular, a standardized method for evaluating the utilization rate of recycled sand in such systems has yet to be established.

This study aims to systematically investigate the effects of incorporating two different particle size ranges of RS (0.075–1.18 mm (RS01) and 1.18–2.36 mm (RS12)) at varying replacement ratios (specifically 100%:0%, 75%:25%, 50%:50%, 25%:75%, and 0%:100%) on the printability of 3D-printed mortar as well as on its hardened mechanical properties, such as compressive strength, flexural strength, interlayer bonding strength. The actual utilization rate of recycled sand will be evaluated using the mass balance method, and the underlying enhancement mechanisms will be elucidated through microstructural analysis.

## 2. Experimental Study

### 2.1. Materials and Mix Proportion Design

**Figure 1:**
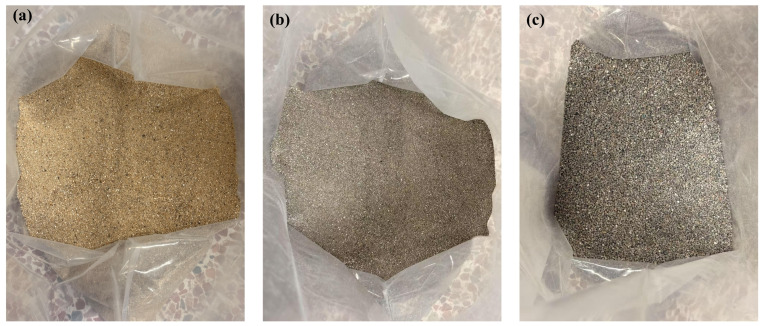
Physical images of the aggregates: (**a**) river sand; (**b**) RS01; (**c**) RS12.

Ordinary Portland cement (P·O 42.5) was used, and its properties are presented in Table 1. As shown in Figure 1, RS with particle sizes of 0.075–1.18 mm and 1.18–2.36 mm was selected as the recycled fine aggregate, while river sand with a particle size of less than 1.18 mm was used as the natural fine aggregate. Their relevant properties are summarized in Table 2. The properties of the silica fume (SF) are provided in Table 3. Polycarboxylate ether superplasticizer (SP) with a water reduction rate of 25% was employed. Sodium gluconate (SG) was employed for setting time control.

As shown in Table 4, six different mix proportions were designed. Two particle sizes of recycled sand were adopted as the main variables. The mass ratios of RS01 to RS12 were 100:0, 75:25, 50:50, 25:75, and 0:100, while the total mass (g) of all mixtures remained constant. The initial mass of cement was fixed at 1000 g, the sand-to-cement ratio was fixed at 1.0, and the water-to-cement ratio was fixed at 0.32. In all mix proportions, SF accounted for 5% of the cement mass to enhance early-age strength and rheological properties. SG was used as a retarder to improve workability retention, while SP was employed to adjust fluidity. The doses of SG and SP were determined based on experimental tests and accounted for 0.12% of the cement weight.

### 2.2. Printing Equipment and Program Settings

**Figure 2:**
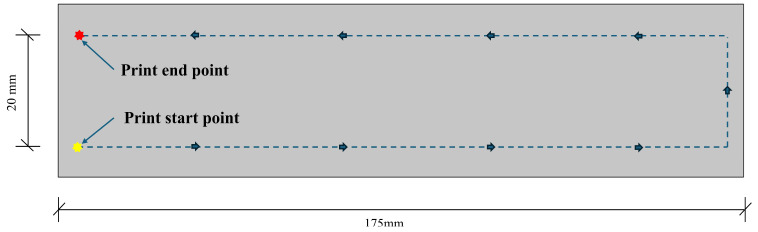
The 3D printing path.

In this study, a concrete (mortar) 3D printer was used. It is a gantry-type system with overall machine dimensions of 1280 mm × 1150 mm × 1400 mm and a printable physical size of 500 mm × 700 mm × 450 mm. The nozzle movement speed and extrusion rate were set to constant values during the printing process. Detailed parameters are provided in Table 5. The printing path shown in Figure 2 was adopted, with a filament spacing of 20 mm. Before printing, the fresh mortar was manually loaded into the hopper, the reference plane was calibrated, and the nozzle was positioned 10 mm above the base plate. The parameters were adjusted according to Table 5, and then the printing process was started.

### 2.3. Specimen Preparation

The mortar was prepared in accordance with GB/T 17671-2021 [29] under controlled environmental conditions (60 ± 5% RH, 25 ± 5 °C). At the beginning of the mixing process, 335 g of water, 1.2 g of SP, and 1.2 g of SG were added to the mixing container. Subsequently, 1000 g of cement and 50 g of SF were introduced, and the mixture was mixed at low speed for 30 ± 1 s. After a pause, 1000 g of sand was uniformly added according to the mix proportion of each group, followed by a low-speed mixing stage of 30 ± 1 s. The mixer was then switched to high speed and mixed for another 30 ± 1 s. The mixing was halted for 90 s, and during the first 15 ± 1 s of this interval, the mortar was scraped from the inner walls of the mixer and gathered back into the container using a trowel. Finally, the mixer was operated at high speed for an additional 60 ± 1 s. After stopping the mixer, the mixed mortar was used to produce specimens.

The cement mortar was cast into molds to prepare cast specimens, which were then placed on a vibrating table to remove air bubbles. Two types of standard specimens were produced: prismatic specimens with dimensions of 40 mm × 40 mm × 160 mm and cubic specimens measuring 40 mm × 40 mm × 40 mm. The 3D-printed specimens had a height of 40 mm, printed in 4 layers. Additionally, 10-layer specimens were printed for buildability testing. After 24 h of printing, all specimens were transferred to a standard curing room maintained for 28 days. After the curing process was completed, the 3D-printed specimens were cut into standard samples and subsequently used for mechanical property testing.

### 2.4. Test Method

This study investigated the printability and mechanical properties of 3D-printable recycled mortar. Flexural and compressive strengths of all cast specimens were tested for comparison. Results show that using recycled sand led to a decrease in mortar strength, consistent with previous research [30,31].

#### 2.4.1. Printability

**Figure 3:**
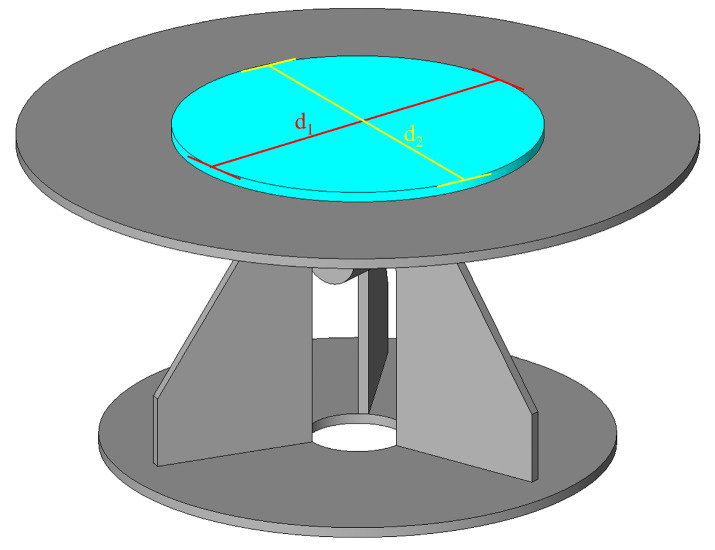
Test method for flowability.

As illustrated in Figure 3, the jump table test was conducted immediately after the mortar mixing was completed, in compliance with GB/T 2419-2005 requirements [32]. The testing procedure involved filling a mold with fresh cement mortar, vertically removing the mold, and subjecting the table to 25 controlled jumps. The resulting mortar spread was measured along two perpendicular diameters (d_1_, d_2_), and the average value was recorded as the flowability. A total of 12 measurements were performed at 10 min intervals.

**Figure 4:**
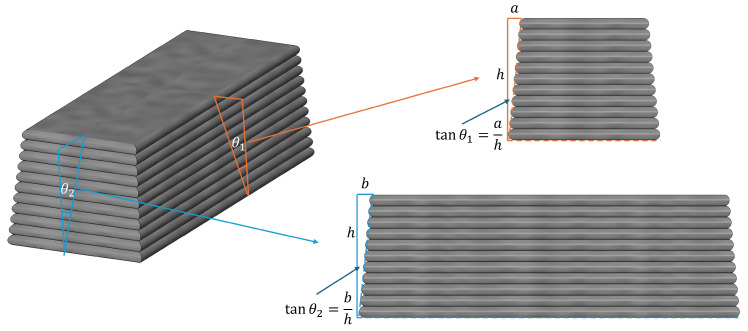
Test method for buildability.

The extrudability was evaluated by observing whether the mortar extrusion process was continuous, free from clogging, and whether the extruded filaments were uniform and smooth. The buildability was assessed through geometric stability tests. As illustrated in Figure 4, the horizontal widths of the top and bottom surfaces, as well as the vertical height of the printed specimen, were measured using a vernier caliper. The layer stability was quantified by calculating the tangent of the deformation angle (θ), derived from the ratio of the width difference to the height; a lower θ value indicates superior buildability [33,34,35], as defined in Equations (1) and (2).

(1) $$\tan\theta_{1}=\frac{a}{h}$$

(2) $$\tan\theta_{2}=\frac{b}{h}$$

#### 2.4.2. Flexural and Compressive Strength Testing

**Figure 5:**
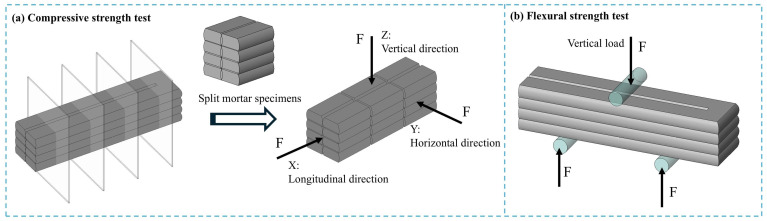
Test methods for compressive and flexural strengths.

As illustrated in Figure 5, flexural strength and compressive strength tests were conducted on the 3D-printed specimens in accordance with GB/T 17671-2021 [29] and GB/T 50081-2019 [36]. The results were compared with those of cast specimens. The 3D-printed filaments were cut into standard dimensions for flexural testing, and the average value of three specimens was recorded. Samples for compressive strength testing were extracted from the 3D-printed filaments along three orthogonal directions: the X-direction (longitudinal), Y-direction (horizontal), and Z-direction (vertical), to evaluate the anisotropic behavior of the printed material. Three designated specimens were tested for each direction, and the average value of the three samples was recorded.

#### 2.4.3. Interlayer Splitting Tensile and Bond Strength Testing

**Figure 6:**
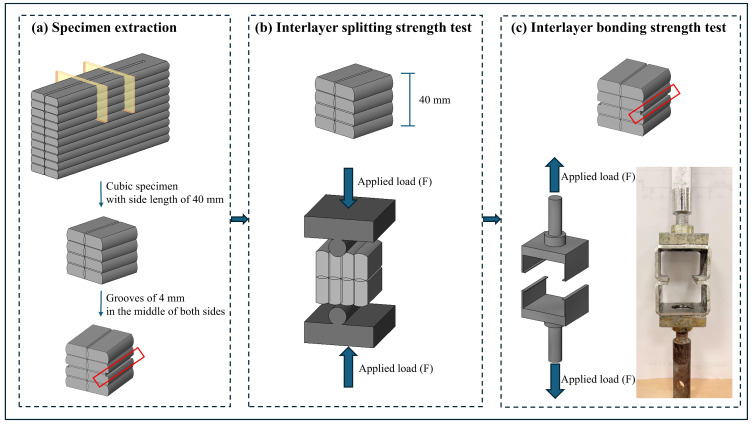
Test methods for interlayer splitting strength and interlayer bond strength.

The interlayer splitting tensile strength and interlayer bonding strength tests were conducted in accordance with the following standards: T/CECS 786-2020 [37], JC/T 2850-2024 [38], and GB/T 50081-2019 [36]. As illustrated in Figure 6, the splitting bearing surfaces of the test specimens were ground to ensure parallelism between the upper and lower surfaces. Each specimen was positioned at the center of the lower platen of the testing machine. Plain round steel bars with a length of 100 mm and a diameter of 18 mm were placed between the specimen and both the upper and lower platens. A loading rate of 0.05–0.08 MPa per second was applied until failure occurred, and the failure load was recorded. Six samples were tested, and the average value was taken. The interlayer splitting strength of the concrete was calculated using Equation (3):

(3) $$f_{ts}=2F/\pi A=0.637F/A$$

where $f_{ts}$ is the splitting tensile strength of concrete (MPa), F is the failure load (N), and A represents the area of the splitting surface (mm^2^).

During the interlayer bond strength test, steel plate molds with a thickness of 10 mm and a side length of 40 mm, featuring 25 mm thick L-shaped extensions on both sides, were placed on the upper and lower surfaces of the specimen. Grooves with a depth of 3 mm and a thickness of 6 mm were made on both sides of the specimen to engage with the corresponding slots in the L-shaped parts of the molds. The average width (b) and length (l) of the fractured interlayer contact surface were measured, and the fracture contact area (A) was calculated as A=b×l. Six samples were tested, and the average value was taken. The interlayer bond strength of the concrete was calculated using Equation (4):

(4) f=F/A  

where f is the splitting tensile strength of concrete (MPa), F denotes the failure load (N), and A represents the interlayer contact area (mm^2^), calculated as A=b×l.

#### 2.4.4. The Material Balance Method

Based on the conceptual approach of the material balance method (bottleneck component method) in chemical reaction engineering, the calculation methodology commences with the known average composition of the raw sand sample, with a defined production target of 1000 g of final product. The net mass of each constituent required was determined according to the designated mix proportion. The source material demand was then traced by reverse-calculating the quantity of raw sand necessary to obtain the aforementioned net masses, based on the content of each component within the raw sand. The bottleneck (identified as the maximum value among all calculated “required raw sand quantities”) dictates the minimum input mass of raw sand essential for producing 1000 g of product, as it is governed by the scarcest component. In principle, the method can be extended to other granular recycled materials when their particle-size distribution and target usable fractions are well defined. The utilization rate is calculated by Equation (5):

(5) $$U=\left(1000\;g\right)/m_{b}$$

where U represents the utilization rate (%), and $m_{b}$ represents the mass of the bottleneck raw material (g).

#### 2.4.5. SEM Testing

The interface morphology characterization and analysis of the heated concrete specimens were carried out using an ultra-high resolution field emission scanning electron microscope. The SEM specimens were square in shape with length and width less than 15 mm and height less than 5 mm, and their surfaces were sputter-coated with a layer of gold.

#### 2.4.6. Vickers Hardness Test

**Figure 7:**
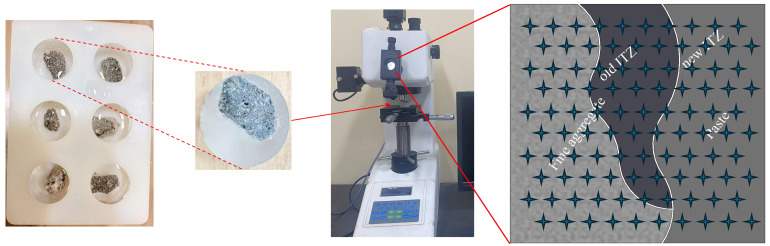
Vickers hardness testing process.

As illustrated in Figure 7, the microhardness and width of the interfacial transition zone (ITZ) of 3D-printed specimens with six different mix proportions were evaluated using an HX-1000T microhardness tester. To ensure a smooth surface of the test specimens, the samples were embedded in epoxy resin and then ground and polished with an angle grinder. A total of 100 measurement points near the ITZ were analyzed, with each point subjected to a 100 g load for 10 s. A 10 × 10 grid of microhardness indentations was made across the interface. The measurement points were spaced 20 µm apart [39,40]. Table 6 summarizes the equipment used in this experiment.

### 2.5. Analysis of Variance

All experimental data were analyzed using one-way and two-way analysis of variance (ANOVA) to evaluate the effects of mix proportion and loading direction on the mechanical properties of 3D-printed mortar. For the analysis of flexural strength, interlayer splitting strength, and interlayer bond strength, a one-way ANOVA was conducted with mix proportions as the sole fixed factor. For compressive strength, a two-way ANOVA was performed with mixed proportions (six levels: R0-0, R1-0, R7-2, R5-5, R2-7, and R0-1) and loading direction (three levels: X-, Y-, and Z-directions) as fixed factors, including their interaction term. The significance level was set at α = 0.05, and post hoc comparisons were performed using Tukey’s honestly significant difference (HSD) test. All statistical analyses were carried out using IBM SPSS Statistics 27.

## 3. Results and Discussion

### 3.1. Utilization Rate of Recycled Sand

Based on the concept of material balance calculation [41,42], the utilization rates of recycled sand under the five mix proportions were calculated. Taking 1000 g of recycled sand as one group (10 groups in total), the proportions of RS01 and RS12 ranges were calculated separately, as shown in Table 7 below. This study addresses the situation where multiple particle sizes coexist in raw recycled sand, while some particle sizes remain unused. Consequently, the “replacement ratio” does not equate to the “actual resource utilization rate”. The material balance method enables the back-calculation of the minimum raw material requirement based on the target particle size demand, thereby better reflecting resource utilization rate.

**Figure 8:**
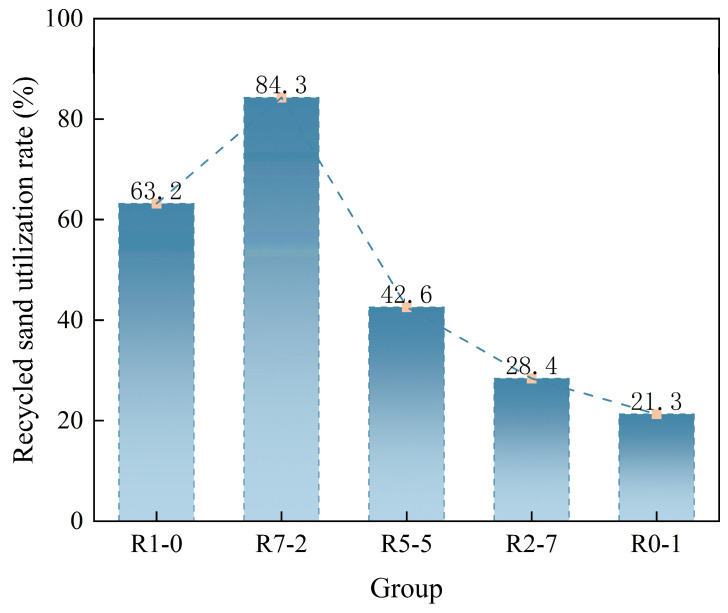
Recycled sand utilization rate.

Under average conditions, for every 1000 g of raw sand, 632 g consists of RS01, 213 g is RS12, and the remaining 155 g comprises other particle sizes (finer or coarser), which were not utilized in this experiment. Targeting the production of 1000 g of blended sand from these two particle size classes, five mix proportions were calculated. For the proportion involving 100% RS01, 1000 g of RS01 was required, necessitating the consumption of 1583 g of raw sand, resulting in a utilization rate of 63.2%. Similarly, under the condition of 75% RS01 and 25% RS12, producing 1000 g of product required 750 g of RS01 and 250 g of RS12. The raw sand quantities needed were 1187 g and 1174 g, respectively. To simultaneously meet both requirements, the maximum value (1187 g) was selected. Recalculating based on this, 750 g of RS01 and 252 g of RS12 were obtained from 1187 g of raw sand, satisfying the requirements. The final utilization rate was calculated as 84.3%. Under the same calculation method, the utilization rates for the other three mix proportions were 42.6%, 28.4%, and 21.3%, respectively. Details are presented in Figure 8.

### 3.2. Flowability

**Figure 9:**
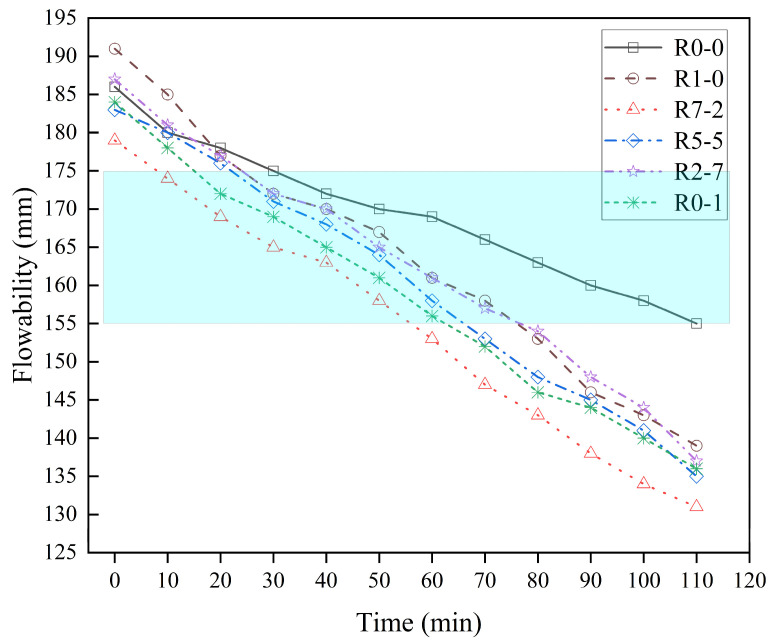
Flowability of the six mortar mixtures.

As plotted in Figure 9, the experimental results indicated that the fluidity of all mortar groups exhibited a gradual decreasing trend within the 120 min observation period, a phenomenon closely related to the cement hydration process and the evaporation and absorption of water in the fresh mortar. Among them, the control group R0-0 showed an initial flow diameter of 186 mm, which eventually decreased to 155 mm, demonstrating a typical time-dependent behavior in fluidity. By comprehensively considering the requirements for printability and structural stability in extrusion-based 3D printing processes, this study identified an optimal fluidity range of 155–175 mm (highlighted in blue in Figure 9) for 3D printing applications. Similarly to findings from other scholarly research, within this range, the mortar possesses sufficient flowability to ensure smooth extrusion while maintaining adequate viscosity to support layer-wise accumulation [43,44].

Due to the higher water absorption and porous structure of recycled sand, the time-dependent loss in fluidity of the mortar groups incorporating recycled sand was significantly more pronounced than that of the natural sand control group [20]. Even after adjustments in the w/c ratio, the fluidity of the recycled sand groups was generally lower than that of the R0-0 group after 20 min. Nevertheless, all recycled sand experimental groups were able to maintain their fluidity within the ideal printable range of 155–175 mm for more than 50 min after mixing, indicating a sufficient time window to meet the demands of practical printing operations. The results demonstrate that through rational mix design and water content management, recycled sands of different particle sizes can be used as aggregates to produce 3D-printable mortars with satisfactory fluidity stability and printing adaptability.

### 3.3. Extrudability

**Figure 10:**
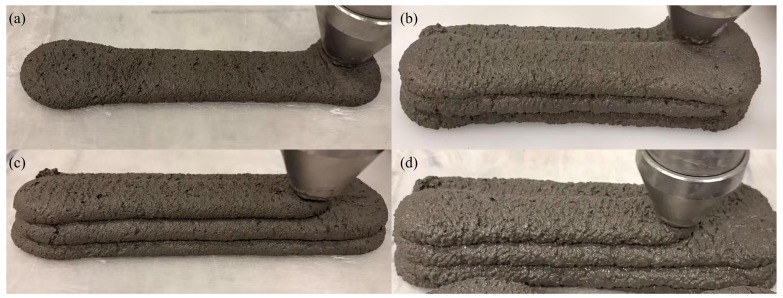
Extrudability of four mix proportions: (**a**) R0-0, (**b**) R1-0, (**c**) R5-5, (**d**) R0-1.

Figure 10 shows that all mortar formulations exhibited favorable extrusion performance. The extruded filaments displayed generally smooth surfaces without significant defects, uniform layer thickness, and well-maintained structural shape, indicating suitable characteristics for 3D printing applications. The R1-0 and R5-5 groups showed homogeneous filament textures with no noticeable coarse aggregate protrusion or segregation, reflecting excellent material compatibility and stable rheological properties. In contrast, the R0-1 group displayed apparent coarse particles distributed on the surface of the extruded filaments, resulting in uneven surfaces. Despite this observable heterogeneity, no extrusion failures (such as fracture, nozzle blockage, or material accumulation) were detected during the printing process. This suggests that, under the specified mix proportion, the incorporation of coarse particles did not significantly compromise extrudability. Within appropriately designed mix proportions, the use of coarse aggregates with suitable particle sizes and incorporation rates may affect the apparent quality and visual uniformity of extruded filaments without critically impairing extrudability. Therefore, subsequent investigations will integrate mechanical tests to comprehensively evaluate the influence of coarse aggregate incorporation on the macro-scale performance of printed components.

### 3.4. Buildability

**Figure 11:**
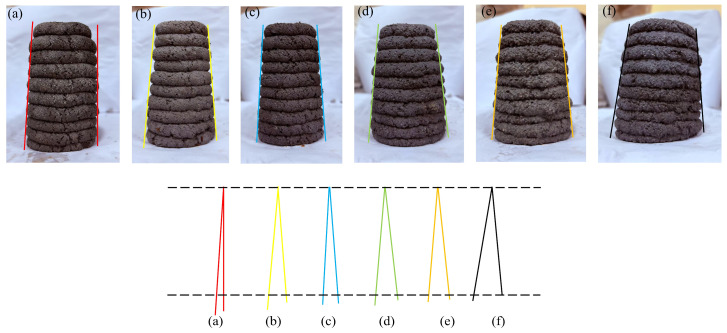
Tilting angle in X-direction: (**a**) R0-0, (**b**) R1-0, (**c**) R7-2, (**d**) R5-5, (**e**) R2-7, (**f**) R0-1.

**Figure 12:**
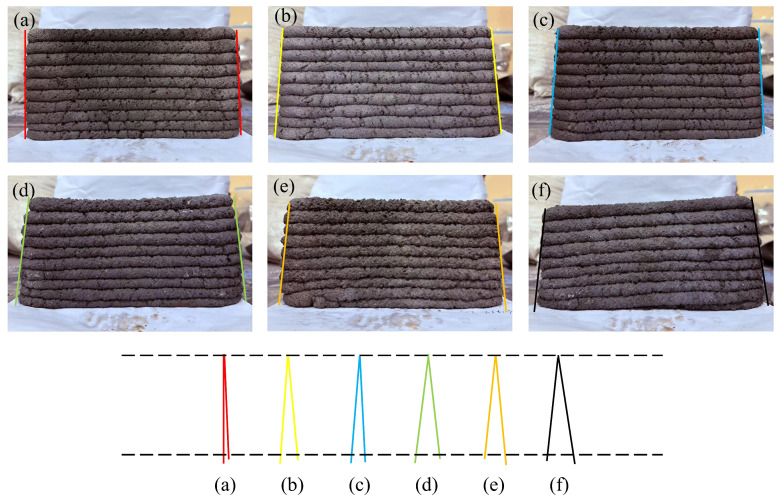
Tilting angle in Y-direction: (**a**) R0-0, (**b**) R1-0, (**c**) R7-2, (**d**) R5-5, (**e**) R2-7, (**f**) R0-1.

In Figure 11, the measured angles from a_1_ to f_1_ are 4.2°, 9.3°, 7.6°, 11.4°, 11.2°, and 15.4°, respectively. In Figure 12, the angles from a_2_ to f_2_ are 3.1°, 9.8°, 7.2°, 12.8°, 11.4°, and 15.1°, respectively. Based on a visual comparison of the angles in both stacking directions, the R0-0 group (natural sand) exhibits the smallest angles, while the R0-1 group shows the largest values. The R1-0 and R7-2 groups also demonstrate relatively favorable performance in terms of stacking angle. Regarding surface characteristics, R0-0 is composed of fine sand with small particle size, displaying fine and smooth texture. In contrast, the experimental groups show progressively increasing uneven surfaces. The R0-0 group exhibits tight interlayer bonding, making it suitable for components requiring smooth surfaces. As the replacement rate of RS12 increases, the surface particle texture becomes more pronounced. The rough surface enhances mortar adhesion, with the R7-2 and R5-5 groups demonstrating improved anti-slip properties and mechanical interlocking, making them applicable for rapid stacking and printing of thick structures. At a 100% replacement rate, the surface is the roughest, with the most noticeable particle presence. The RS12 particles result in significant surface unevenness.

The buildability of the specimens from groups R0-0, R1-0, R7-2, R5-5, R2-7, and R0-1 was quantitatively evaluated using Equation (1) by calculating tanθ. The computed values of $\tan\theta_{1}$ and $\tan\theta_{2}$ are summarized in Table 8. The control group R0-0 showed the smallest tanθ values, indicating the best buildability. Among the experimental groups, R7-2 exhibited the smallest tanθ values (0.066 and 0.063), demonstrating excellent buildability, followed by the R1-0 group with values of 0.082 and 0.086. The R0-1 group yielded the highest tanθ values (0.136 and 0.133), reflecting relatively poor buildability. The magnitude of the tan value reflects the excellence of its buildability, which is consistent with the findings reported by numerous scholars [45,46].

Among the experimental groups, the stacking angle exhibited an initial decrease, followed by a plateau, and then a subsequent increase. This behavior originates from the self-weight effect of the mortar during the layer-by-layer printing process. The progressive accumulation of height increases the gravitational load, subjecting the lower layers to cumulative compressive stress, which subsequently induces deformation. These variations can be explained by the combined effects of particle size distribution, paste rheology, and load-bearing capacity.

When RS01 was used, the high viscosity of the paste resulted in significant extrusion resistance and strong interlayer bonding, which reduced the tendency for slumping or deformation. However, the load-bearing capacity of the bottom layers was relatively poor, and the shape retention ability after extrusion was limited, thereby restricting the maximum printable height. As the content of RS12 particles increased, a stable “skeleton structure” was formed. This structure enhanced load-bearing capacity and shape stability while promoting faster strength development. Simultaneously, the improved fluidity of the paste and sufficient interlayer bonding provided mechanical support to the printed layers, enabling them to resist deformation and sustain the weight of subsequent layers. In contrast, when only RS12 particles were used, the lack of fine sand led to increased and enlarged voids. Consequently, the paste was unable to effectively encapsulate and bind the aggregates, which resulted in potential separation between the mortar and aggregates during extrusion. This, in turn, weakened the interlayer bonding and contributed to a rough extruded surface.

### 3.5. Compressive Strength

**Figure 13:**
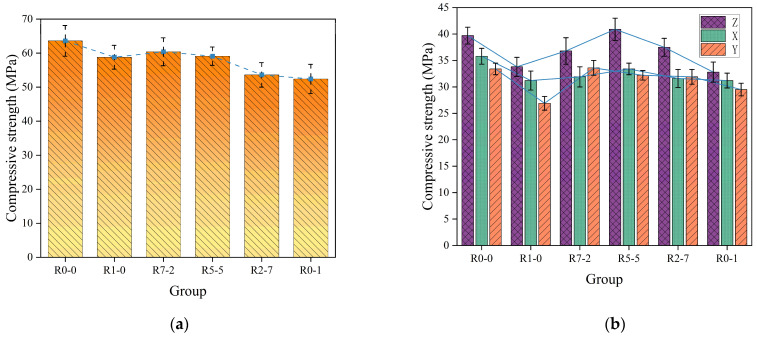
Compressive strength: (**a**) Cast specimens, (**b**) 3D-printed specimens.

In Figure 13a, the compressive strength of cast specimens ranged from 52.4 to 63.6 MPa. The control group R0-0, prepared with natural sand, exhibited the highest compressive strength of 63.6 MPa, followed by groups R7-2 and R5-5 with strengths of 60.4 MPa and 59.1 MPa, respectively. In Figure 13b, for the 3D-printed specimens, the compressive strength of the natural sand control group R0-0 varied between 33.4 MPa and 39.7 MPa across the X, Y, and Z directions. Among the experimental groups, the compressive strength in the three directions ranged from 26.9 MPa to 40.9 MPa. The strength in the Z-direction consistently reached the highest values, while the values along the X- and Y-directions exhibited significant variability. Cast specimens exhibit higher compressive strength than 3D-printed specimens, which is consistent with the findings of many scholars [47,48,49].

From R1-0 to R0-1, the compressive strength along the X, Y, and Z directions initially increased and then decreased with the increasing proportion of RS12. This trend aligns with that observed in the cast specimens. As shown by the SEM images in Section 3.9, an appropriate amount of RS12 optimizes the continuous gradation of the mortar, reducing the amount of cement paste required to fill the voids. This results in a denser microstructure and lower porosity of the mortar matrix, thereby directly enhancing its compressive strength. However, compared with natural sand, recycled aggregate inherently possesses higher porosity, lower density, and an existing old interfacial transition zone (ITZ) [22,30]. Consequently, when RS12 is used as a complete replacement for natural sand, the newly formed ITZ between the aggregate and the fresh mortar matrix is intrinsically weaker than that in natural aggregate-based mixtures, which macroscopically manifests as a reduction in overall mechanical performance.

The experimental results showed that the highest average compressive strength was observed in the Z-direction, whereas the lowest average compressive strength was found in the X- and Y-directions. When loaded in the Z-direction, the applied stress was perpendicular to the printed layers. The extrusion pressure exerted on the material during the printing process induced a compaction effect in the vertical direction, leading to denser interlayer bonding and fewer defects in the Z-direction. In contrast, the lower strength in the X- and Y-directions can be attributed to the minimal pressure experienced by the horizontal direction during the setting process. Furthermore, the horizontal direction was jointly affected by the interface between adjacent printing filaments, interlayer voids, and incomplete contact. These defects are more likely to act as crack initiation sites and propagation paths when the material is loaded horizontally [50].

### 3.6. Flexural Strength

**Figure 14:**
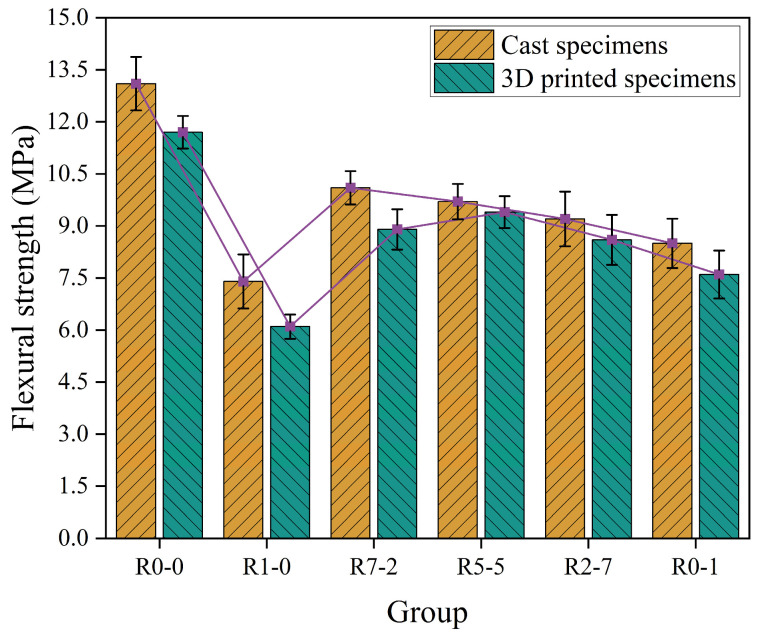
Flexural strength.

As illustrated in Figure 14, the flexural strength values of the group R0-0 were measured at 11.7 MPa and 13.1 MPa, respectively. Among the experimental groups, R7-2 and R5-5 exhibited relatively better flexural performance, with values of 10.1 MPa and 8.9 MPa for R7-2, and 9.7 MPa and 9.4 MPa for R5-5. The flexural strength of cast specimens was higher than that of their 3D-printed counterparts. The corresponding strength reductions were 10.7%, 17.6%, 11.9%, 3.1%, 6.5%, and 10.6%, respectively. Cast specimens were prepared by placing mortar into standard molds followed by thorough vibration on a shaking table, resulting in a homogeneous, continuous, and nearly isotropic internal structure. In contrast, 3D-printed specimens were fabricated through layer-by-layer deposition via extrusion from a printhead. This process leads to anisotropic strength properties. Compared to conventional vibration-compaction methods, the printing process lacks effective compaction to eliminate air bubbles. As a result, the printed structures contain more and larger pores, particularly in the interlayer regions, where air entrapment often occurs, leading to microvoids and defects that significantly compromise the structural integrity of the material [51].

In group R1-0, the uniformity in RS01 resulted in numerous voids after stacking, creating multiple weak points in the structure. Under loading, cracks readily initiated and propagated at these defects. In group R5-5, with an increased replacement rate of RS12, the coarse particles formed a skeletal structure, while the fine particles effectively filled the interparticle voids, leading to a denser overall microstructure with fewer defects. However, in group R0-1, the amount of fine sand was insufficient to fully encapsulate and fill the gaps between coarse particles. A significant volume of voids had to be filled solely by cement paste, resulting in numerous large internal defects. Cracks easily initiated at the weak interfaces between coarse particles and the cement matrix and propagated rapidly, leading to premature failure. Recycled sand with different particle sizes yields distinct effects [52]. This explains the observed trend of flexural strength initially increasing and then decreasing with higher coarse sand content.

**Figure 15:**
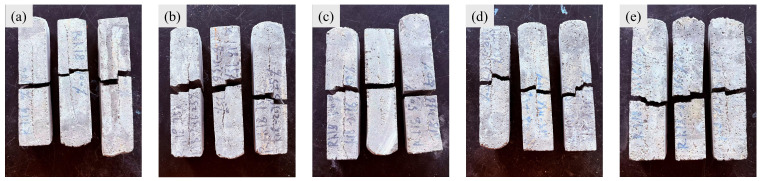
Physical image of flexural test specimens. (**a**) R1-0, (**b**) R7-2, (**c**) R5-5, (**d**) R2-7, (**e**) R0-1.

As shown in Figure 15, the fracture surfaces of the recycled sand experimental groups also exhibited rough and irregular morphologies, predominantly characterized by zigzag patterns, exhibiting obvious brittle failure [53]. The overall fracture process resulted from the alternating propagation of cracks in both horizontal and vertical directions. At the midspan of the bottom surface (tensile side), where the tensile stress reached its maximum, cracking initiated at the weakest regions, typically the interlayer interfaces. Since the interlayer bond strength (tensile/shear strength) was considerably lower than that of the bulk mortar, the crack initially propagated along the X-Y plane. When the horizontally propagating crack encountered an obstacle or experienced a change in the stress state at its tip, it tended to seek the path of least resistance, often altering its direction to extend vertically upward (or downward) through the mortar matrix toward an adjacent interlayer interface. Upon reaching a new interlayer, the crack resumed horizontal propagation. This process repeated cyclically: horizontal extension along an interlayer → vertical penetration through a mortar layer → horizontal extension along the subsequent interlayer.

### 3.7. Interlayer Bond Strength

**Figure 16:**
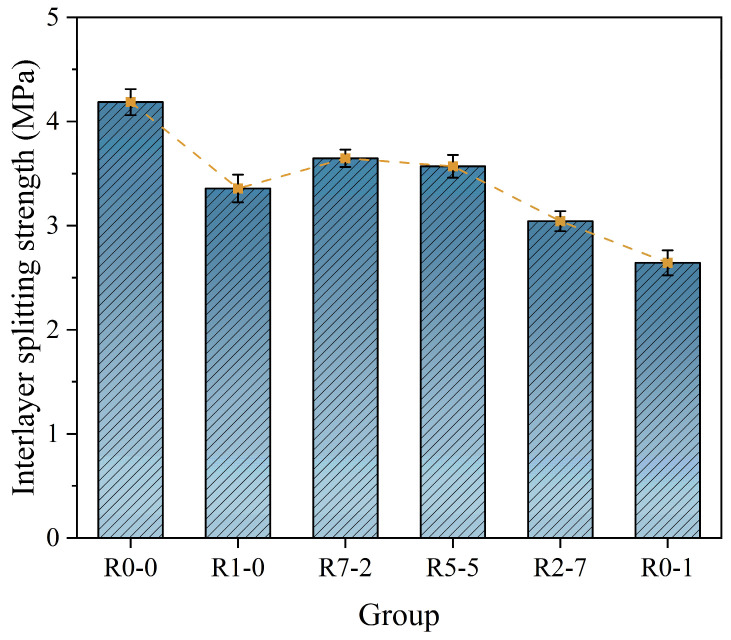
Interlayer splitting strength.

**Figure 17:**
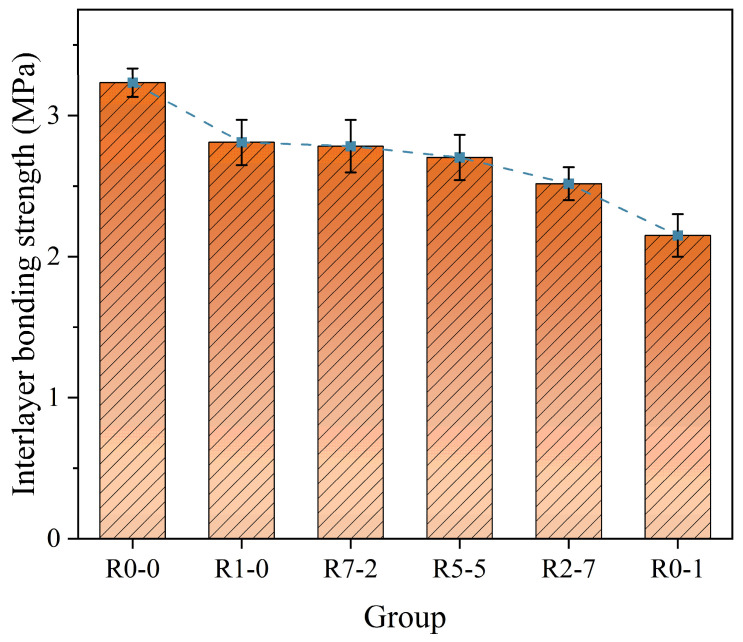
Interlayer bonding strength.

As illustrated in Figure 16 and Figure 17, the control group (R0-0) exhibited significantly higher interlayer strength compared to the experimental groups, which can be attributed to the superior inherent properties of the natural sand material. In group R1-0, the uniform particle distribution at the interface resulted in the weakest mechanical interlocking effect [54], with strength relying on limited bonding from hydration reactions. When a small amount of RS12 was added, a mechanical interlock formed between the coarse and fine particles, effectively increasing the contact area and reducing interfacial defects between the aggregates. Meanwhile, the RS01 particles filled the voids, resulting in a denser interface. Failure under load likely occurred partially at the interface and partially within the bulk material, indicating enhanced mechanical anchoring.

From R1-0 to R0-1, the interlayer bond strength initially remained stable and then exhibited a declining trend [55]. When only RS01 was used, the mortar demonstrated favorable fluidity, resulting in tighter interlayer contact and effective mechanical interlocking as well as chemical bonding. With the incorporation of RS12, while fine sand remained dominant, the mixture maintained good chemical bonding performance, and the mechanical interlocking effect was further enhanced.

However, when the proportion of RS12 became excessively high (e.g., R7-2 and R5-5), localized voids or defects were observed at the interface [56]. The increased presence of porous and thin-walled regions reduced the effective load-bearing area. Additionally, the mortar exhibited increased roughness and reduced contact area between layers, leading to the formation of noticeable voids and defects that acted as weak points in the structure. Furthermore, the workability of the mortar (including extrudability and buildability) was compromised, adversely affecting compaction quality during layer deposition. Mechanistically, a moderate increase in surface unevenness may enhance mechanical interlocking, whereas excessive unevenness reduces the effective contact area between adjacent layers and promotes interfacial voids, thereby weakening interlayer bonding.

### 3.8. Analysis of Variance of Mechanical Properties

Table 9 presents the results of the two-way ANOVA for compressive strength. The analysis indicated that mix proportion (F = 26.88, *p* < 0.001), loading direction (F = 52.28, *p* < 0.001), and their interaction (F = 5.97, *p* < 0.001) all exerted statistically significant effects on compressive strength. The effect sizes (η^2^) revealed that mix proportion and loading direction accounted for 64.7% and 52.4% of the total variance, respectively, demonstrating that both are key factors influencing compressive strength. Furthermore, the significant interaction effect (*p* < 0.001) indicated that the influence of loading direction on compressive strength was not uniform across different mix proportions; rather, the degree of anisotropy varied depending on the specific RS12/RS01 ratio employed.

**Figure 18:**
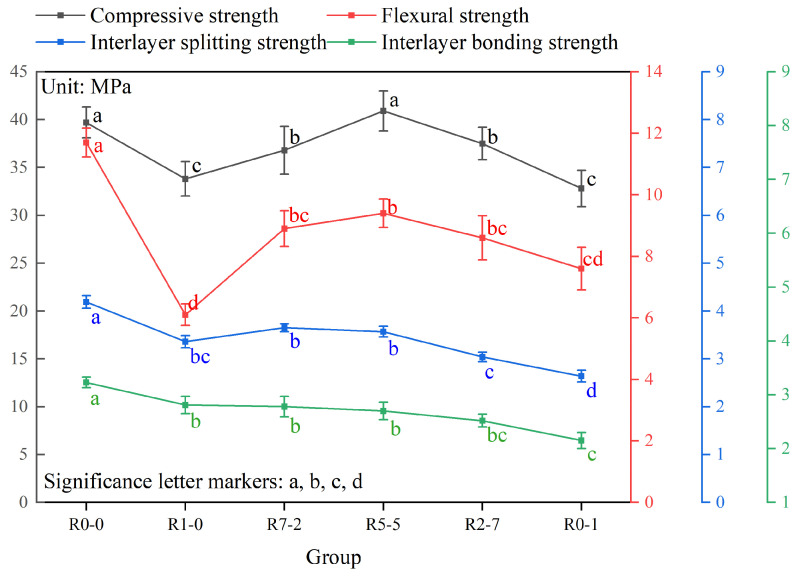
Different letters indicate significant differences.

Table 10 presents the one-way ANOVA results for flexural strength, interlayer splitting strength, and interlayer bond strength. The analysis showed that mix proportion exerted a highly significant effect on all three mechanical properties (*p* < 0.001). The partial eta-squared (η^2^) values all exceeded 0.92, demonstrating that mix proportion accounts for over 90% of the variance in each property, thereby serving as the predominant factor determining these mechanical properties. This finding underscores that adjustments to the RS12/RS01 ratio were highly effective in modulating the mechanical performance of 3D-printed recycled mortar. To further identify which specific mix proportions differed from one another, post hoc comparisons were conducted using Tukey’s HSD test. The results of these pairwise comparisons are denoted by letters in Figure 18.

Different letters indicate statistically significant differences among groups (*p* < 0.05), while the same letter denotes no significant difference (*p* > 0.05), based on Tukey’s HSD test. R0-0 represents natural sand control; R1-0, R7-2, R5-5, R2-7, and R0-1 represent recycled sand mixtures with varying RS12/RS01 ratios. Tukey’s HSD post hoc analysis showed that the R5-5 and R0-0 mixtures yielded significantly higher compressive strengths than the other groups (*p* < 0.05). For loading direction, the Z-direction gave the highest value (36.92 MPa), followed by the X-direction (32.50 MPa) and Y-direction (31.25 MPa), with all pairwise differences being significant (*p* < 0.05). A significant interaction effect was observed, indicating that the degree of anisotropy depended on the mix proportion. Regarding flexural strength, the R5-5 mixture (9.40 MPa) outperformed the R1-0 (6.10 MPa) and R0-1 (7.60 MPa) mixtures (*p* < 0.05). For interlayer splitting strength, the R7-2 (3.65 MPa) and R5-5 (3.57 MPa) mixtures showed significantly higher values than the R2-7 (3.04 MPa) and R0-1 (2.64 MPa) mixtures (*p* < 0.05). Interlayer bond strength decreased with increasing RS12 content, with that of the R0-1 mixture (2.15 MPa) being significantly lower than those of the other recycled sand groups (*p* < 0.05).

### 3.9. SEM Analysis

**Figure 19:**
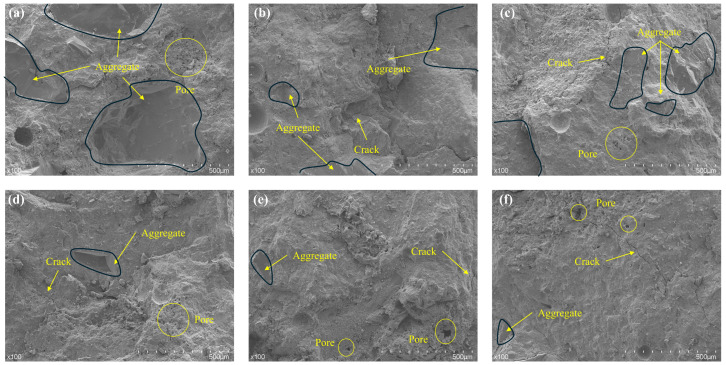
SEM image at 100× magnification: (**a**) R0-0, (**b**) R1-0, (**c**) R7-2, (**d**) R5-5, (**e**) R2-7, (**f**) R0-1.

In Figure 19, irregularly shaped aggregate particles, ranging in size from several tens to hundreds of micrometers, can be clearly observed forming the fundamental skeleton of the material. In Figure 19a, the aggregate particles are primarily interconnected by the surrounding cement paste. In Figure 19b–f, the influence of aggregate gradation variation on the microstructure is evident. Figure 19b shows that the 0–1.18 mm fine aggregates are bonded by cement paste. This structure is prone to stress concentration at the paste interfaces under loading, leading to the propagation of microcracks along the brittle interfaces and around the fine aggregate particles, thereby limiting the mechanical performance of the material. In Figure 19c–e, the coarse aggregates established mechanical interlocking between coarse particles through fine aggregates and cement paste, forming a continuous three-dimensional mechanical network that significantly enhanced load transfer capacity. The coarse aggregates served as the primary load-bearing skeleton, effectively distributing external stress, while the fine aggregate–paste composite bridging zones provided secondary support with stiffness and strength substantially higher than those of pure cement paste. Additionally, flocculent silica fume agglomerates were uniformly dispersed within the paste, filling the gaps between fine aggregates or adhering to particle surfaces, further improving the density of the interfacial transition zone. When microcracks extended into the bridging regions, they were forced to deflect, bypass, or branch, thereby enhancing the toughness and crack resistance of the material.

However, when only coarse aggregates were used (Figure 19f), the lack of multi-scale particle gradation hindered the formation of an effective bridging network. The aggregates were connected solely by cement paste, which merely coated the surfaces of the coarse particles. In this case, microcracks tended to propagate directly along the interfacial transition zones, resulting in degraded mechanical properties.

**Figure 20:**
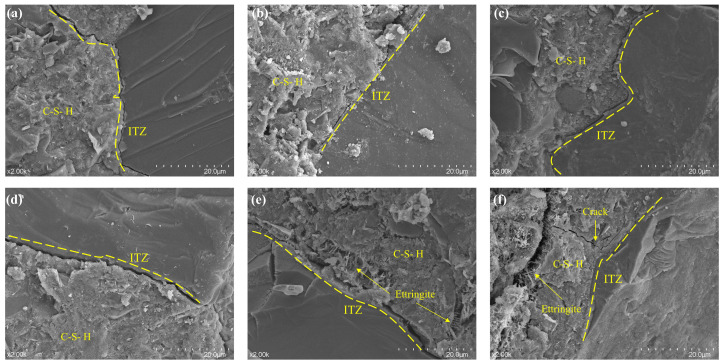
SEM image at 2000× magnification: (**a**) R0-0, (**b**) R1-0, (**c**) R7-2, (**d**) R5-5, (**e**) R2-7, (**f**) R0-1.

As shown in Figure 20, distinct pores, hexagonal plate-like CH crystals, and microcracks propagating along the interfaces were frequently observed in the ITZs. In contrast, following the incorporation of silica fume, the ITZs between coarse/fine particles and the cement paste in the bridging regions became remarkably thin and dense. A substantial amount of amorphous C-S-H formed a continuous and uniform coating, firmly bonding the aggregate particles to the paste matrix. The number of hexagonal CH crystals significantly decreased, and their size was refined [22]. Moreover, these crystals were enveloped by C-S-H, effectively mitigating their weakening effect on the interface. Compared to samples with solely fine or coarse aggregates, high-magnification observations revealed that the gaps between coarse and fine aggregates were not filled merely by cement paste alone. Instead, they formed composite bridging systems with fine aggregates as the core, densely encapsulated by C-S-H [57].

In contrast, Figure 20a reveals that the natural sand particles exhibited smooth surfaces and distinct ITZs yet lacked such multi-scale particle synergies to enhance the microstructure. In Figure 20b–d, the C-S-H gel not only filled pores but also penetrated the microscopic defects of coarse and fine particles, creating strong mechanical interlocking. The additional C-S-H generated by the pozzolanic reaction of silica fume significantly enhanced the chemical bonding between aggregates and the paste. The crack widths in the ITZs of groups with optimized gradation were narrower than those in Figure 20e,f. The synergistic effect of improved particle packing and silica fume effectively suppressed the initiation and propagation of interfacial cracks.

### 3.10. Vickers Hardness Analysis

**Figure 21:**
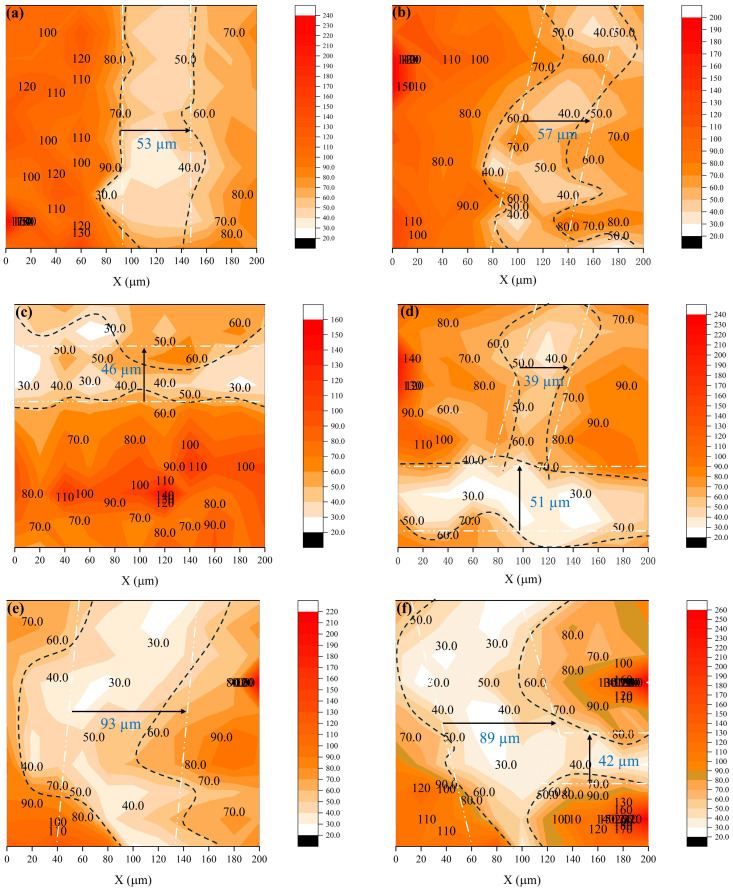
Microhardness distribution of ITZ: (**a**) R0-0, (**b**) R1-0, (**c**) R7-2, (**d**) R5-5, (**e**) R2-7, (**f**) R0-1.

Figure 21 presents microhardness profiles near the ITZ for different mixed proportions. The ITZ width was estimated based on the distinct hardness differences observed among the aggregate, the ITZ, and the bulk paste. Points of sharp hardness change were used to delineate iso-hardness contours, and the average width was taken as the ITZ width. The measured widths in Figure 21a–f are 53 µm, 57 µm, 46 µm, 51 µm, 93 µm, and 89 µm, respectively. Additionally, bifurcation of the ITZ, as shown in Figure 21d,f, is considered to result from the interaction between new and old ITZs. As the proportion of RS12 increases, the ITZ width initially decreases to a smaller value but subsequently increases, accompanied by more pronounced interfacial effects.

In the case of the R1-0 mix, the uniform fine sand particles led to a single-sized particle packing structure with significant voids. Although the water-to-cement ratio remained constant, the poor packing density likely resulted in higher overall porosity within the microstructure. Additionally, the RS01 particles tended to align preferentially along the interface, further increasing porosity. These factors collectively contributed to a weaker and wider new–old ITZ. When the proportion of RS12 was excessively high, the ITZ surrounding individual coarse aggregate particles became broader and more porous. Even when microhardness indentation was intentionally performed away from the aggregate, the indentation points were more likely to fall within the influenced zone of the ITZ, leading to lower measured hardness values. Moreover, during the 3D printing process, the coarse sand particles were more prone to segregation, causing paste enrichment and aggregate deficiency at the interlayer interface. This phenomenon formed a weak paste-rich layer, further compromising the ITZ quality.

The optimally graded mixes (R7-2 and R5-5) maximized the mechanical anchoring advantage provided by the coarse sand particles, while avoiding the issues of interfacial porosity caused by excessive fine sand and segregation induced by excessive coarse sand. These balanced proportions facilitated effective bridging across new and old interfaces, disrupting the continuity of the weak ITZ pathway. Consequently, the most compact and robust new–old ITZ was formed, which was directly reflected in the minimal ITZ width observed in these mixes [58,59,60].

## 4. Conclusions

The printability of the 3D-printed mortar incorporating two sizes of recycled sand was investigated by evaluating its buildability and flowability. The mechanical properties were also evaluated, while the microstructure was characterized using SEM and microhardness analysis. The main conclusions are as follows:

(1) Based on the concept of the limiting component in chemical process design and through material balance calculations, the maximum utilization rate of recycled sand reached 84.3% when the RS12/RS01 particle ratio was 1/3.

(2) All five groups of recycled mortar exhibited excellent flowability and extrudability. When the RS12/RS01 particle ratio was 1/3, the tilt angles in bidirectional buildability were the smallest, measuring 7.6° and 7.2°, with tanθ values of 0.066 and 0.063, respectively, demonstrating outstanding buildability.

(3) When the RS12/RS01 particle ratio was 1/3, the compressive strength, flexural strength, and interlayer splitting strength increased by an average of 36.5%, 40.7%, and 6.8%, respectively, compared to the mortar with only fine particles. When the ratio was 1/1, the strengths increased by an average of 31.1%, 41.5%, and 5.3%, respectively. The interlayer bonding strength remained relatively consistent.

(4) ANOVA revealed that mix proportion and loading direction significantly affect the mechanical properties of 3D-printed recycled mortar (*p* < 0.001). Mix proportion explained over 92% of the variance in flexural, interlayer splitting and bond strength, while loading direction accounted for 52.4% of the variance in compressive strength.

(5) The packing of different RS12/RS01 particles enhanced load transfer, which strengthened the chemical bonding between the aggregates and cement paste. The effect of particle packing effectively suppressed the development of interfacial cracks. The microhardness results indicate that the packing effect of RS12 and RS01 contributed to a reduction in the width of the ITZ. When the ratio was 1/3, its interfacial transition zone reached a minimum of 46 μm.

It should be noted that the findings of this study are limited to the utilization of recycled sand derived from waste concrete within two specific particle size ranges due to limitations of the experimental equipment. Future research will aim to extend the scope of application by incorporating other types of waste materials (e.g., masonry, ceramics and glass, stone, pure mortar, and even asphalt and lightweight materials), exploring a wider range of particle sizes, and further considering the effects of curing methods, age, and environmental factors (e.g., carbonation, high temperature, freeze–thaw, wetting–drying, and their coupling effects) on durability.

## Figures and Tables

**Table 1:** Cement performance indicators.

Test	Compressive Strength (MPa)		Flexural Strength (MPa)		Setting Time (min)	
	3d	28d	3d	28d	Initial Setting Time	Final Setting Time
Standard value	≥17.0	≥42.5	≥3.5	≥6.5	≥45	≤600
Tested value	18.2	49.8	4.6	7.9	137	426

**Table 2:** Physical properties of fine aggregate.

Fine Aggregate	Apparent Density (kg/m^3^)	Water Absorption (%)	Fineness Modulus	Crushing Value (%)
River sand	2580.0	2.9	2.43	14.5
Recycled sand	2158.6	24.8	4.16	25.7

**Table 3:** Chemical composition of silica fume.

Chemical Composition	CaO	SiO_2_	Al_2_O_3_	Fe_2_O_3_	SO_3_	Others
Mass fraction (%)	1.43	96.22	0.71	0.25	0.31	1.08

**Table 4:** Mix proportion design of 3D-printed recycled mortar (g).

Group	Cement	SF	River Sand 0.075–1.18 mm	Recycled Sand 0.075–1.18 mm	Recycled Sand 1.18–2.36 mm	SG	SP	Water
R0-0	1000	50	1000	0	0	1.2	1.2	335
R1-0	1000	50	0	1000	0	1.2	1.2	355
R7-2	1000	50	0	750	250	1.2	1.2	355
R5-5	1000	50	0	500	500	1.2	1.2	355
R2-7	1000	50	0	250	750	1.2	1.2	355
R0-1	1000	50	0	0	1000	1.2	1.2	355

Note: “R” denotes RS. The first number indicates the first digit of the incorporation ratio (%) of recycled sand with a particle size smaller than 1.18 mm, while the second number represents the first digit of the incorporation ratio (%) of RS with a particle size ranging from 1.18 mm to 2.36 mm.

**Table 5:** Parameters of the 3D printer.

Parameters	Print Length (mm)	Extrusion Width (mm)	Single Layer Height (mm)	Nozzle Diameter (mm)	Moving Speed (mm/s)	Extrusion Speed (r/s)	Print Accuracy (mm)
Value	175.0	20.0	10.0	20.0	50.0	1.2	1.0

**Table 6:** Equipment list.

Equipment Name	Model	Manufacturer	City	Country
Laboratory-grade concrete (mortar) 3D printer	GL-3DPRT-L	Hangzhou Guanli Intelligent Technology Co., Ltd.	Hangzhou	China
Cement mortar mixer	JJ-5	Wuxi Jianyi Experiment Instrument Co., Ltd.	Wuxi	China
Apparatus of fluidity of cement mortar	NLD-3	Wuxi Jianyi Experiment Instrument Co., Ltd.	Wuxi	China
Microhardness tester	HX-1000T	Shanghai Yuguang Instrument Co., Ltd.	Shanghai	China
Ultra-high resolution field emission scanning electron microscope	Regulus 8100	Hitachi High-Tech Corporation	Tokyo	Japan
Microcomputer-controlled electro-hydraulic servo universal testing machine	WAW-600C	Jinan Shijin Instrument Co., Ltd.	Jinan	China

**Table 7:** Proportions of recycled sand with different particle sizes.

Group	1	2	3	4	5	6	7	8	9	10
0.075–1.18 mm	620	648	640	628	621	632	622	631	637	641
1.18–2.36 mm	225	207	209	208	213	204	209	212	216	224
Others	155	145	151	164	166	164	169	157	147	135

**Table 8:** tanθ values for different mix proportions.

*θ*	R0-0	R1-0	R7-2	R5-5	R2-7	R0-1
$\tan\theta_{1}$	0.037	0.082	0.066	0.101	0.098	0.136
$\tan\theta_{2}$	0.028	0.086	0.063	0.112	0.101	0.133

**Table 9:** Results of two-way ANOVA on compressive strength.

Source of Variation	Sum of Squares	df	Mean Square	f-Value	*p*-Value	η^2^
Mix proportion	330.90	5	66.18	26.88	<0.001	0.647
Loading direction	257.43	2	128.72	52.28	<0.001	0.524
Mix proportion × Loading direction	147.09	10	14.71	5.97	<0.001	0.422

**Table 10:** Results of one-way ANOVA on mechanical strength.

Mechanical Properties	Index	Sum of Squares	df	Mean Square	F-Value	*p*-Value	η^2^
Flexural strength	Between-group variance	57.40	5	11.48	30.09	<0.001	0.926
	Within-group variance	4.58	12	0.38	/	/	/
Interlayer splitting strength	Between-group variance	6.36	5	1.27	94.44	<0.001	0.929
	Within-group variance	0.49	12	0.04	/	/	/
Interlayer bonding strength	Between-group variance	2.75	5	0.55	28.97	<0.001	0.923
	Within-group variance	0.23	12	0.02	/	/	/

## Data Availability

The original contributions presented in this study are included in the article. Further inquiries can be directed to the corresponding author.
